# Local Backbone Geometry Plays a Critical Role in Determining Conformational Preferences of Amino Acid Residues in Proteins

**DOI:** 10.3390/biom12091184

**Published:** 2022-08-26

**Authors:** Nicole Balasco, Luciana Esposito, Alfonso De Simone, Luigi Vitagliano

**Affiliations:** 1 Institute of Molecular Biology and Pathology, CNR c/o Dep. Chemistry, University of Rome Sapienza, P.le A. Moro 5, 00185 Rome, Italy; 2 Institute of Biostructures and Bioimaging, CNR, Via Pietro Castellino 111, 80131 Naples, Italy; 3 Department of Pharmacy, University of Naples Federico II, 80131 Naples, Italy

**Keywords:** propensity scales, NC^α^C angle, secondary structure, (φ, ψ) torsion angles, Ramachandran plot

## Abstract

The definition of the structural basis of the conformational preferences of the genetically encoded amino acid residues is an important yet unresolved issue of structural biology. In order to gain insights into this intricate topic, we here determined and compared the amino acid propensity scales for different (φ, ψ) regions of the Ramachandran plot and for different secondary structure elements. These propensities were calculated using the Chou–Fasman approach on a database of non-redundant protein chains retrieved from the Protein Data Bank. Similarities between propensity scales were evaluated by linear regression analyses. One of the most striking and unexpected findings is that distant regions of the Ramachandran plot may exhibit significantly similar propensity scales. On the other hand, contiguous regions of the Ramachandran plot may present anticorrelated propensities. In order to provide an interpretative background to these results, we evaluated the role that the local variability of protein backbone geometry plays in this context. Our analysis indicates that (dis)similarities of propensity scales between different regions of the Ramachandran plot are coupled with (dis)similarities in the local geometry. The concept that similarities of the propensity scales are dictated by the similarity of the NC^α^C angle and not necessarily by the similarity of the (φ, ψ) conformation may have far-reaching implications in the field.

## 1. Introduction

Proteins are biomolecules characterized by extraordinary molecular and structural complexities that are crucial for their functional roles. In proteins, the structural complexity is coupled with a remarkable fine regulation. Indeed, although proteins may be tolerant to amino acid substitutions, frequently even the replacement/introduction of very few non-hydrogen atoms may completely undermine their structure/activity [1]. The combination of complexity and fine regulation makes a full understanding of protein structural properties and propensities extremely difficult. The decoding of the so-called folding code represents a central issue in structural biology [2,3]. In its general definition, the folding code underlies three distinct aspects related to protein structures: (a) the folding pathway (kinetic of the folding), (b) the stability of the folded state compared to the unfolded ones (thermodynamics of the folding) and (c) the three-dimensional structure of the folded state (protein structure prediction) [2,3].

The seminal experiment of Anfinsen [4] that established an intimate link between protein sequence and its three-dimensional structure represents one of the founding events of structural biology. This observation has been translated in the so-called Anfinsen postulate stating that the structure of a protein is dictated by its amino acid sequence. Although many limitations and exceptions of this principle have been highlighted over the years, its validity is generally accepted by the structural biology community. In this scenario, a corollary of the Anfinsen postulate is that a protein structure should be determined from its sequence. After decades of unsuccessful attempts [5] or limited success (Rosetta) [6], some impressive achievements have been obtained in recent years with the application of the machine learning approaches implemented in the AlphaFold algorithm [7,8]. It is noteworthy that these approaches, being based on artificial intelligence, do not provide clues on the physicochemical basis of the folding process. Therefore, we are currently inundated by a large amount of accurately predicted structural data still ignoring many basic principles governing protein structures.

The sequence–structure relationship established by the Anfinsen postulate also implies that different types of protein residues must have different conformational preferences. The distinct residue preference for specific conformations or structural elements has been demonstrated for decades by developing propensity scales using either experimental or statistical approaches [9,10,11,12,13,14,15,16,17,18,19,20,21]. Although a general consensus between these scales has been assessed [9], the structural and physicochemical bases underlying this observation are still discussed. Indeed, despite the many hypotheses put forward over the years [10,19,20], it is not clear, with the exception of Gly and Pro residues that present unique structural features, what dictates the preference of residues for either α-helical regions or β-sheet structures. However, it is important to note that protein residues are in fact not endowed with stringent conformational preferences since each of them can be accommodated in a variety of structural elements and can stably φ and ψ values in various regions of the Ramachandran plot. None of the common secondary structure motifs is precluded to any of the protein-encoded amino acid residues. Indeed, their conformational versatility allows the generation of a huge number of distinct structural motifs and folds with a relatively limited number of building blocks (twenty amino acid types). This versatility makes the unraveling of the basis of residue propensities a rather difficult puzzle whose solution is of fundamental importance for protein engineering and design.

This fundamental issue was here approached by an analysis of the conformational preferences of residues for specific regions of the Ramachandran plot [22,23], a fundamental tool widely used in structural biology for protein structure analysis and validation [24,25,26]. In particular, we dissected the Ramachandran plot in 15° × 15° (φ, ψ) boxes and for each box, a statistical propensity scale following the Chou–Fasman definition [27], which is based on the frequencies of each residue in the box, was generated. Then, adjacent boxes were clustered if they presented similar scales in order to identify Ramachandran regions with homogenous propensity scales. This procedure led to the identification of seven distinct clusters whose propensity scales were then calculated and compared. The comparison of the propensity scales of these regions unraveled unexpected and interesting correlations and anticorrelations. Moreover, we observed that (dis)similarities of propensity scales between different regions of the Ramachandran plot are coupled with (dis)similarities in the local geometry. These findings clarified the crucial role that local geometry plays in determining conformational preferences of amino acid residues in protein structures.

## 2. Materials and Methods

### 2.1. Selection of the Protein Structures

Statistical surveys were performed on a dataset of non-redundant (sequence identity ≤ 25%) protein chains selected from the Protein Data Bank (PDB) (release of April 2015). The choice to use this release of the PDB was dictated by the consideration that in recent years some refinement programs [28] use conformation-dependent geometry for restraints. Since the conformational-dependent geometry variability is an important aspect of this work (see results), we preferred to use an unbiased ensemble of PDB structures. These chains were also sorted from protein structures (chain length ≥ 40 residues) solved at a resolution better than 2.2 Å and refined to an R-factor lower than 0.20 (Data2.2). The PDB codes of the 5566 non-redundant and well-refined protein structures (5766 protein chains) selected and some experimental parameters (chain length, resolution, R-factor) are reported in Table S1a. As in our analyses, we selected only atoms identified in the PDB files with the ATOM card, modified residues whose atoms were labeled as HETATM were not considered (e.g., selenomethionine). On the other hand, residues whose side chain was modified but retained the ATOM labeling were considered for further selections (e.g., glycosylation, phosphorylation). The statistical analyses were carried out for residues whose average backbone B-factor (atomic displacement parameter) was lower than 1.3 times the average backbone B-factor of their own chain (B-factor selection). The elimination of a residue on the basis of the B-factor prevents the possibility of calculating (φ, ψ) angles for the adjacent ones, which were therefore also excluded in this study. This selection led to an ensemble of 5439 protein chains (Table S2a) containing 1,089,468 residues.

A higher resolution ensemble (Data1.6, Table S1b) was used to calculate the value of the τ (NC^α^C) angle. In particular, 2658 PDB entries (2731 protein chains) were selected using the following criteria: resolution better than 1.6 Å, sequence identity ≤ 25%, and R-factor ≤ 0.20. The B-factor selection led to an ensemble of 2612 protein chains (Table S2b).

The dataset of 2967 π-helices identified by Karplus PA [29] and colleagues in over 2400 non-redundant (<90% sequence identity) protein chains was used in the analysis of the conformation adopted by residues in π-helices. From this initial ensemble, a smaller one (1970 protein chains) was derived by considering only π-helices of seven residues. This database includes 2288 π-helices. The conformation (φ, ψ angles) of the residues which occupy the seven different positions was analyzed.

### 2.2. Sets of Protein Residues

This study has been performed considering different ensembles of amino acid residues. Since the side chains of many residues are able to directly interact with the local backbone through hydrogen bonding and/or electrostatic or aromatic interactions thus affecting the local conformation of the residue, most of the analyses were performed considering a subset, denoted as 9AA, of nine residues (A, V, L, I, M, K, R, Q, E) that includes those whose side-chains cannot establish electrostatic, polar, or π-type interactions with the local backbone atoms. Along this line, we selected residues with totally apolar aliphatic side chains (A, V, L, I). We also included in this ensemble those amino acid residues whose polar, charged or aromatic groups could not interact with their own backbone atoms. In particular, we considered residues whose functional group was beyond the C^γ^ (M, K, R, Q, E). These analyses were also extended on a set of the eighteen non-Pro/non-Gly residues, which includes all protein encoded residues, with the exception of Gly and Pro, which display peculiar and well-documented behaviors. This set of residues was denoted as 18AA ensemble. Considering the protein ensemble of Data2.2, the 9AA and 18AA datasets contain 582,585 and 965,557 amino acid residues, respectively.

### 2.3. Definition of Statistical Propensities

The assignment of the most common secondary structure elements such as α-helix (H), 3(10)-helix (G) and β-sheet (E) was performed using DSSP [30,31]. The assignment of polypropline II (P) fragments was performed by adopting the criteria reported by Berisio et al. [32]. Statistical propensities for specific secondary structure elements were calculated using the Chou–Fasman definition [27] (1):

(1) $$P_{X,j}=\left(\frac{N_{X,j}}{N_{tot,j}}\right)/\left(\frac{\sum_{i=1}^{20}N_{X,i}}{\sum_{i=1}^{20}N_{tot,i}}\right)$$

where *N_X,j_* is the number of the *j* residue adopting the *X* conformation (E, H, G or P) while *N_tot,j_* is the total number of the *j* residue in the dataset. This ratio is normalized by considering the 20 amino acid residues commonly found in proteins.

Chou–Fasman-like propensities were also computed for specific (φ, ψ) boxes of the Ramachandran plot. In detail, the (φ, ψ) Ramachandran space was divided into 576 (φ, ψ) 15° × 15° square boxes identified by a number (1 to 24 for −180° < φ < 180°) and a letter (A to X for −180° < ψ < 180°). The population of these boxes in terms of the number of residues is reported in the Supplementary Figure S1. The propensity of a certain residue for a specific box was calculated using the following definition (2):

(2) $$P_{box,j}=\left(\frac{N_{box,j}}{N_{tot,j}}\right)/\left(\frac{\sum_{i=1}^{20}N_{box,i}}{\sum_{i=1}^{20}N_{tot,i}}\right)$$

where *N_box,j_* is the number of the *j* residue in a given (φ, ψ) box while *N_tot,j_* is the total number of the *j* residue in the dataset. An example of the calculation is shown in the Supplementary Figure S2.

A Chou–Fasman-like approach was also applied to calculate the propensities to occupy a specific position (denoted as *PIk*, *k* = 1 to 7) of the π-helix (3):

(3) $$P_{PIi,j}=\left(\frac{N_{PIk,j}}{N_{tot,j}}\right)/\left(\frac{\sum_{i=1}^{20}N_{PIk,i}}{\sum_{i=1}^{20}N_{tot,i}}\right)$$

where *N_PIk,j_* is the number of the *j* residue that occupies the *PIk* position while *N_tot,j_* is the total number of the *j* residue in the dataset. Propensity scales were generated by calculating and ranking the propensities for all the amino acid residues of the ensemble.

### 2.4. Evaluation of the Statistical Significance of the Results

Similarities between propensity scales of different (φ, ψ) regions were evaluated by linear regression analyses in terms of the correlation coefficient R. The significance of the correlation coefficients between different scales was established with the so-called null hypothesis. The statistical test yields a *p*-value which represents the probability that random sampling would result in a correlation coefficient as far from zero as observed in our dataset, under the hypothesis that there is no correlation between the two variables; *p*-values < 0.01 or <0.001 allow one to reject the null hypothesis at the 99% or 99.9% confidence level, respectively.

## 3. Results and Discussion

### 3.1. Selection of PDB Structures and Definition of Residue Subsets

The analysis of the conformational preferences of the protein residues was performed by selecting high-resolution and well-refined structures reported in the PDB. Applying the criteria reported in the Methods sections we selected 5766 non-redundant protein chains (Data2.2). These structures were inspected to remove mobile residues on the basis of their B-factor values (see Methods). Most of the analyses have been conducted on the set of nine amino acid residues (9AA ensemble) whose aliphatic side chains do not possess functional groups (A, V, L, I) and those whose functional group was beyond the C^γ^ atom (M, K, R, Q, E). The findings obtained using the 9AA were then evaluated by considering the eighteen non-Pro/non-Gly residues (18AA ensemble).

### 3.2. Identification of Ramachandran Plot Regions with Homogenous Statistical Propensity Scales

**Figure 1:**
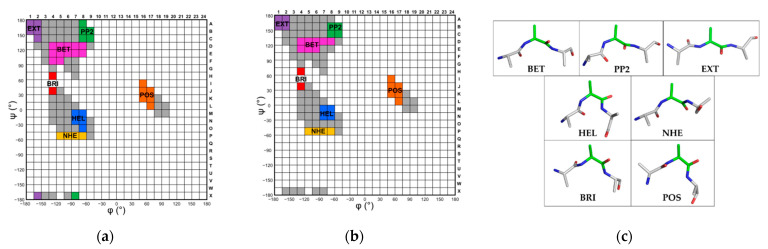
The 95 (φ, ψ) boxes (15° × 15°) containing at least 1000 residues in Data2.2 are colored. Adjacent boxes displaying similar propensity scales of (**a**) the 9AA (R > 0.8, *p*-value < 0.01) or (**b**) the 18AA (R > 0.6, *p*-value < 0.01) were clustered and depicted with the same color. Boxes whose propensity scales do not correlate with the others are in grey. The notation of the clusters is explained in the main text. (**c**) Stick representation of an alanine tripeptide with the central residue adopting (φ, ψ) dihedral angles corresponding approximately to the center of the different clusters identified in the Ramachandran plot: BET (−105°, 120°), PP2 (−60°, 150°), EXT (−165°, 165°), HEL (−75°, −15°), NHE (−90°, −67.5°), BRI (−127.5°, 60°), and POS (60°, 30°).

The large amount of structural data available in the Protein Data Bank (PDB) makes the calculation of statistical (Chou–Fasman-like) propensity scales possible even for small regions of the Ramachandran plot. In this scenario, we dissected the (φ, ψ) Ramachandran space in 576 (φ, ψ) square boxes (15° × 15°) identified by a number (1 to 24 for −180° < φ < 180°) and a letter (A to X for −180° < ψ < 180°) (e.g., 3A, 4F, etc.). Then, we preliminarily evaluated the population of these boxes (Figure S1) and considered only those containing more than 1000 residues for the subsequent analyses (light grey boxes in Figure S1). The populations of these boxes are also reported in Supplementary Table S3 (total number of the 20 amino acid residues) and S4a (number of each type of residue). For each of the 95 highly-populated boxes Chou–Fasman-like (φ, ψ) propensity scales were calculated as detailed in the Methods section (Supplementary Figure S2 and Table S4b). In order to identify regions of the Ramachandran space characterized by homogenous residue propensities, these boxes were clustered on the basis of the similarities of the propensity scales. In particular, for each couple of boxes, the propensity scales of the 9AA were compared by performing a linear regression analysis (see as example Supplementary Figure S3 for the comparison of the pairs 6D-6E and 6D-6C). A box was included in a cluster if the correlation coefficient of its propensity scale with those of all the other members of that cluster was higher than 0.80 (*p*-value < 0.01). This procedure led to the identification of seven distinct clusters (Figure 1a).

The inspection of Figure 1 clearly indicates that Ramachandran plot regions underlying well-defined structural motifs (α-helices and β-sheets) are indeed somehow inhomogeneous when analyzed in terms of conformational preferences of amino acid residues. In particular, the region classically associated with extended conformations (−180° < φ < −45° and ψ > 90°) reveals a break into three distinct clusters. In addition to the regions corresponding to the well-known β-strand and polypropline II conformations (denoted as BET and PP2, respectively), a third region with distinct conformational preferences has been identified. This cluster, denoted as EXT, includes residues adopting highly extended conformations (−180° < φ < −150° and ψ > 135°) (Figure 1).

Even more surprising is the observation that in the region corresponding to helical states two clusters endowed with distinct propensity scales could be detected (Figure 1a). In addition to the canonical helical conformation (denoted as HEL), which is characterized by (−90° < φ< −60°) and (−45° < ψ < 0°), a distinct cluster characterized by more negative values of both φ and ψ has been found (denoted as NHE, new helical region) (Figure 1).

Finally, two additional clusters characterized by similar values of the ψ (0° < ψ < 60°) but either negative or positive values of the φ angle have been identified. These states, denoted as BRI and POS, correspond to the bridge region and to the α_L_ conformation, the latter characterized by positive values of the φ angle, respectively (Figure 1). Similar results were obtained by comparing the propensity scales of the 18AA (Figure 1).

### 3.3. Comparisons of the Propensity Scales of the Clusters: Correlations and Anticorrelations

Once we assessed the occurrence and the extension in the Ramachandran space of seven regions with homogenous propensity scales, we systematically compared them by performing a linear regression analysis on all pairs of clusters (Table 1).

**Figure 2:**
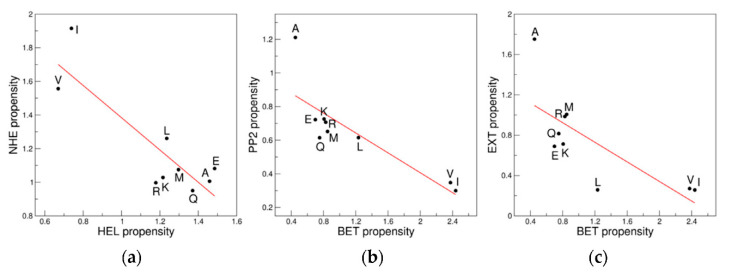
Anticorrelations of the propensity scales of proximal clusters: (**a**) HEL *versus* NHE; (**b**) BET *versus* PP2; and (**c**) BET *versus* EXT. The 9AA ensemble is considered.

Interestingly, this analysis unraveled previously undetected correlations/anticorrelations among the propensity scales computed for the 9AA ensemble of distant regions of the Ramachandran plot. Intriguingly, proximal clusters often show anticorrelated scales whereas the scales of very distant clusters may be correlated. Anticorrelations are indeed observed for regions that may be associated with the same secondary structural element. In particular, the proximal clusters in the helical region, HEL and NHE, exhibit significantly anticorrelated scales (R = −0.86, *p* = 0.003) (Figure 2a). Similarly, extended conformations that are assumed by residues populating BET, PP2 and EXT also exhibit anticorrelations. Indeed, the propensity scale of the BET region anticorrelates with that of the PP2 (R = −0.83, *p* = 0.005) and EXT (R = −0.74, *p* = 0.022) clusters (Figure 2b,c).

**Figure 3:**
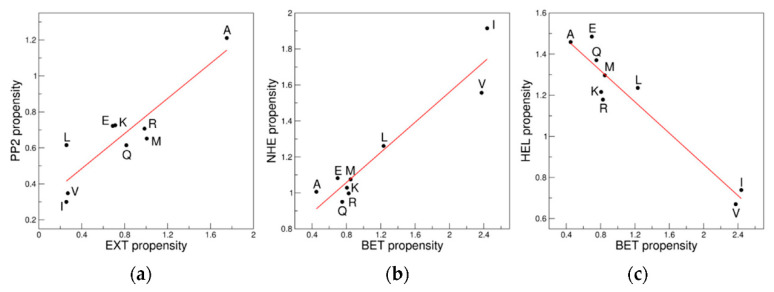
Examples of correlation/anticorrelations of the propensity scales of distant clusters: (**a**) EXT *versus* PP2; (**b**) BET *versus* NHE; and (**c**) BET *versus* HEL. The 9AA ensemble is considered.

Among distant regions highly significant correlations are shown by PP2 and EXT (R = 0.90, *p* = 0.001) (Figure 3a). More interestingly, BET correlates with NHE (R = 0.95, *p* = 9.1 × 10^−5^) and anticorrelates with HEL (R = −0.96, *p* = 4.7 × 10^−5^) (Figure 3b,c). Significant, although lower, correlations/anticorrelations are detected for PP2 *versus* HEL (R = 0.79, *p* = 0.011) and for POS *versus* NHE (R = −0.82, *p* = 0.007). The BRI cluster shows less significant correlations with the other clusters. Barely significant correlations/anticorrelations are observed with POS (R = 0.72, *p* = 0.029) and NHE (R = −0.70, *p* = 0.036).

The analysis of the data reported in Table 1 indicates that five clusters (EXT, BET, PP2, HEL, NHE), which correspond to the highest populated regions of the Ramachandran plot, may be divided into two larger groups: (i) G1 made of EXT, HEL, and PP2 and; (ii) G2 made of BET and NHE. These groups have the following properties: (1) the propensity scales of the members of each group are significantly correlated; and (2) the scales of clusters belonging to the two groups anticorrelate (*p* ranging from 0.048 to 4.7 × 10^−5^). The analysis of the propensity scales of the remaining clusters POS and BRI suggests that they may be associated with the G1 group, although the correlations are not always statistically significant. As mentioned above, the trends highlighted on the reduced set of nine residues were evaluated considering the eighteen non-Pro/non-Gly protein residues (Table 2).

Although the overall picture of correlations/anticorrelations illustrated in the previous paragraph is complicated by the presence of functional groups on the side chains of the other nine residues (D, N, C, H, W, T, S, Y, and F), the trends detected for the 18AA ensemble resemble those detected for the 9AA selection (Table 1 and Table 2). Indeed, if we compare the values of the correlation coefficients (R values) that emerged for the regression analyses on the same pair of clusters considering either the 9AA or the 18AA ensemble a significant correlation emerges (R = 0.94, *p* < 10^−5^—Supplementary Figure S4a). The two groups of points in the plot represent correlations (positive R values) and anticorrelations (negative R values) between the different clusters identified in the Ramachandran plot.

**Figure 4:**
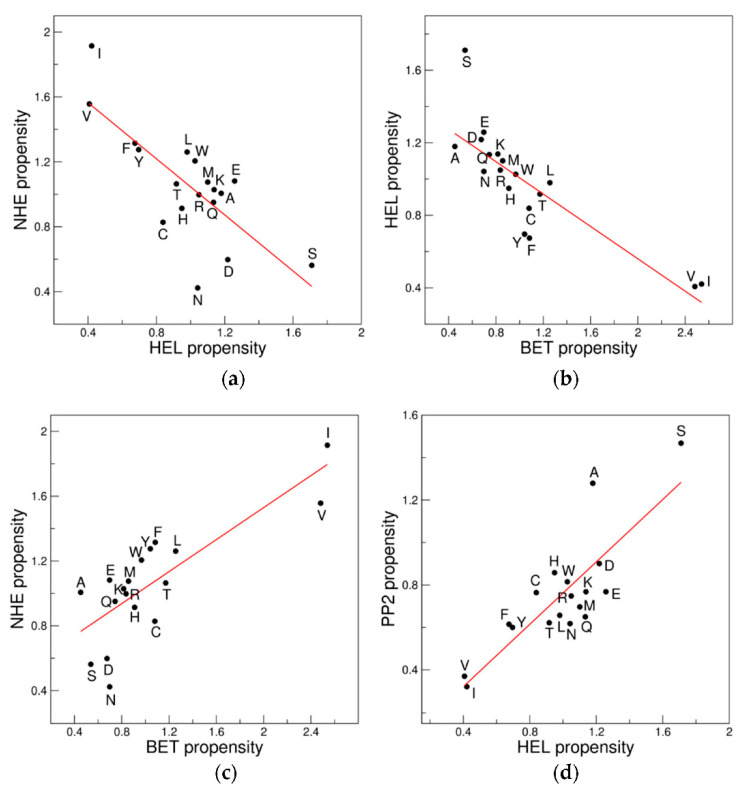
Examples of correlation/anticorrelations of the propensity scales of the clusters: (**a**) HEL *versus* NHE; (**b**) BET *versus* HEL; (**c**) BET *versus* NHE; and (**d**) HEL *versus* PP2. The 18AA ensemble is considered.

Particularly evident are the anticorrelations observed with the 18AA ensemble for the regions HEL-NHE and BET-HEL as well as the correlations of BET-NHE and HEL-PP2 (Figure 4).

Moreover, to further evaluate the impact of local side chain–main chain interactions on the propensity scales, we also computed correlations/anticorrelations for the ensemble (Other9AA) composed of the other nine residues (D, N, C, H, W, T, S, Y, and F) whose side chain could interact with the backbone by making electrostatic, polar or aromatic contacts (Table 3). Compared to 9AA, for this ensemble, we observe smoothed correlation/anticorrelation patterns when evaluated in terms of R values (Figure S4b). However, although the presence of specific functional groups has an impact on these propensity scales, some observations are also preserved in the Other9AA ensemble. In particular, the propensity scales of PP2 and HEL are significantly correlated (R = 0.88, *p* = 0.002). On the other hand, significant anticorrelations could be observed for BET *versus* PP2 (R = −0.88, *p* = 0.002), BET *versus* HEL (R = −0.81, *p* = 0.009), and HEL *versus* NHE (R = −0.74, *p* = 0.022) (see Table 3 for further details).

Collectively, these findings shed light on previously undetected correlations/anticorrelations between the propensity scales of distant regions of the Ramachandran plot.

### 3.4. The Newly Identified NHE Region Is Overpopulated by Residues Found in π-Helices

As highlighted in the previous sections, the clustering of (φ, ψ) boxes displaying homogenous propensity scale organizations leads to the identification of well-defined regions in the Ramachandran plot. Some of these regions may be straightforwardly associated with well-known secondary structure elements (α-helix, 3 (10)-helix, β-sheet, and polypropline II). On the other hand, the NHE cluster, although close to the helical region of the plot, is characterized by (φ, ψ) torsion angles that are significantly different from those corresponding to the canonical α-helix (−63°, −43°). A survey of literature data suggests that the (φ, ψ) values of the NHE region resemble those associated with the structure of the π-helix [29,33,34]. It has been shown that this structural motif is evolutionarily derived from the insertion of a single residue into an α-helix leading to the i, i + 5 hydrogen bonding pattern that defines the π-helix [29]. Using the dataset of seven-residue π-helices identified by Karplus PA and colleagues [29] (see Methods for details), we analyzed the conformation (φ, ψ angles) adopted by residues at each position (denoted as PIk, k = 1 to 7) of the helix (Supplementary Figure S5). The average (φ, ψ) values are reported in Table S5. These data show that residues located at the positions PI5 and PI6 of π-helices adopt the here-defined NHE conformation (Figure S5 and Table S5 in Supplementary Material). Therefore, the NHE region is able to accommodate the α-helix distortions that lead to the formation of the π-helix.

**Figure 5:**
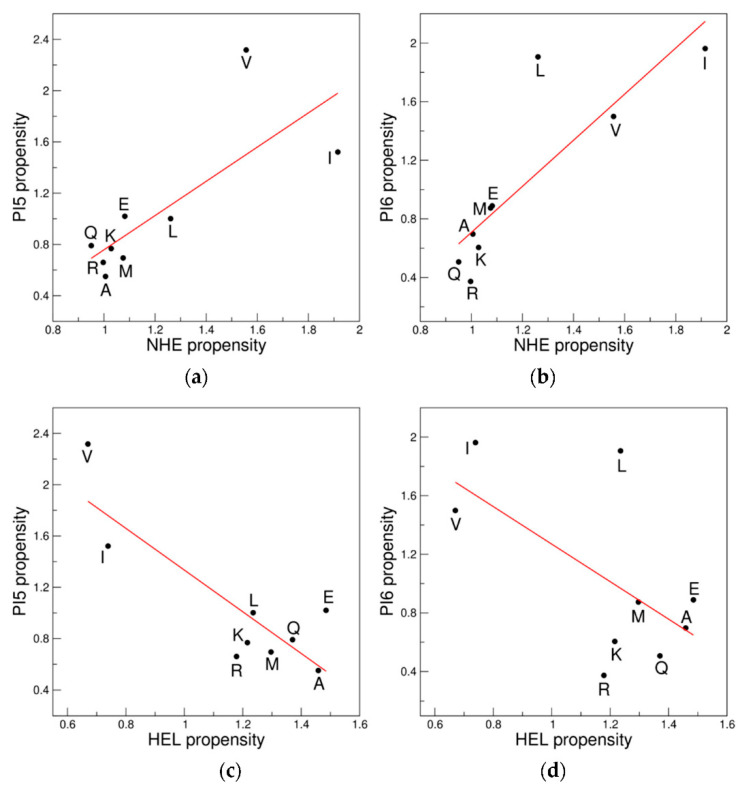
Correlations of the propensity scales for NHE/HEL and positions five (PI5) or six (PI6) of π-helices: (**a**) NHE *versus* PI5; (**b**) NHE *versus* PI6; (**c**) HEL *versus* PI5; and (**d**) HEL *versus* PI6. The 9AA ensemble is considered.

In order to validate this result, we defined and calculated for each amino acid residue the Chou–Fasman-like propensity to occupy a specific position of the π-helix (see Methods for details). This approach allowed us to define propensity scales for the seven positions that were then compared with the scales of NHE and HEL. As expected, significant correlations were detected in the 9AA ensemble between the NHE propensity scale and the scales of positions five PI5 (R = 0.77, *p* = 0.014) and six PI6 (R = 0.85, *p* = 0.0039) (Figure 5a,b). As a consequence, the scales of these positions anticorrelate with the propensity scale of HEL: HEL *versus* PI5 (R = −0.85, *p* = 0.0039), HEL *versus* PI6 (R = −0.62, *p* = 0.075) (Figure 5c,d). Moreover, when the 18AA ensemble is considered, the correlations/anticorrelations are still significant: NHE *versus* PI5 (R = 0.55, *p* = 0.018), NHE *versus* PI6 (R = 0.81, *p* = 5.0 × 10^−5^), HEL *versus* PI5 (R = −0.71, *p*= 9.8 × 10^−4^), HEL *versus* PI6 (R = −0.47, *p* = 0.048).

Finally, it is important to note that NHE boxes present a relatively low population if compared to the boxes of the HEL region (Figure S1). This is due to the fact that in regions characterized by negative φ values a progressively increasing repulsion between the C^β^ and the H_(i+1)_ atoms occurs when ψ values decrease [35]. Therefore, structural states characterized by the regular repetition of this conformation are rare, but they may represent a common deformation of the α-helix (for example helix aneurism). Nevertheless, the drastic difference in amino acid residue preferences for the contiguous regions HEL and NHE is a remarkable finding.

### 3.5. Propensity Scales for Secondary Structure Elements: Correlations and Anticorrelations

**Figure 6:**
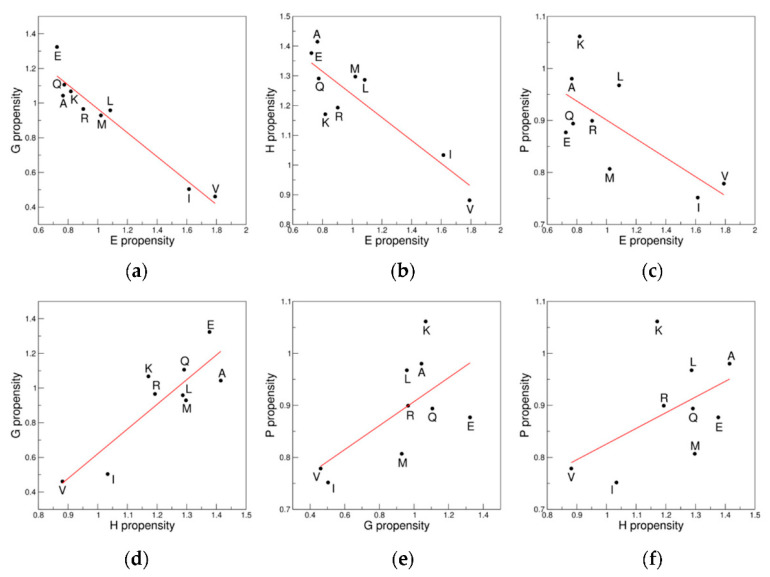
Correlation/anticorrelations of the propensity scales for specific secondary structure elements: (**a**) E *versus* G; (**b**) E *versus* H; (**c**) E *versus* P; (**d**) H *versus* G; (**e**) G *versus* P; and (**f**) H *versus* P. The 9AA ensemble is considered.

It is worth noting that the conformation of residues belonging to secondary structure elements frequently spans large areas of the Ramachandran plot. This is particularly evident for the β-sheet structural motif. Moreover, secondary structure residues, while forming the hydrogen-bond pattern of the motif, may display conformations with significant deviations from the canonical ones due to local distortions or terminal effects. This is evident for the α-helix that occasionally presents distortions such as alpha-aneurysm [29,34]. Therefore, residues of secondary structure elements, although located in specific regions, may present a significant dispersion in the Ramachandran plot. In particular, residues belonging to a specific secondary structure element may fall in distinct clusters in the Ramachandran plot and, conversely, the same cluster may contain residues adopting different secondary structures (see also below). We here developed and analyzed propensity scales based on the frequency of the different residues to adopt a secondary structure element rather than a specific (φ, ψ) region (see Methods for the definition). Again, the initial investigations were performed considering the reduced 9AA ensemble. These analyses clearly indicate that the propensity scales for different secondary structure elements may be significantly correlated/anticorrelated (Table 4 and Figure 6). In particular, we observed that the propensity scale for the β-structure (E) anticorrelates with those of the other elements: 3 (10)-helix (G), α-helix (H), and polypropline II (P) (Figure 6a–c). Notably, the E *versus* G is remarkable (R = −0.96, *p* = 3.5 × 10^−5^). Significant correlations are detected for the pair-wise comparison of the G, H, and P scales (Figure 6d–f).

**Figure 7:**
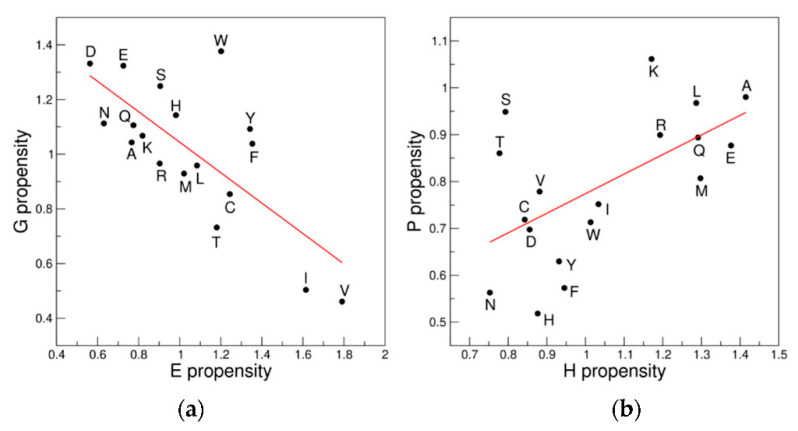
Significant correlation/anticorrelations of the propensity scales detected in the 18AA ensemble: (**a**) E *versus* G; and (**b**) H *versus* P.

The extension of these analyses to the 18AA ensemble only partially confirms the trends observed for 9AA (Supplementary Table S6). Of interest is the previously undetected in literature anticorrelation between the E and G scales (R = −0.73, *p* = 6.1 × 10^−4^) (Figure 7a). A significant correlation is also observed between the H and P scales (R = 0.59, *p* = 0.01) (Figure 7b). Our analysis of the propensity scales for specific secondary structure elements also highlights previously unidentified correlations/anticorrelations.

### 3.6. (φ, ψ) Versus Secondary Structure Scales: The Propensity/Structure Puzzle

**Figure 8:**
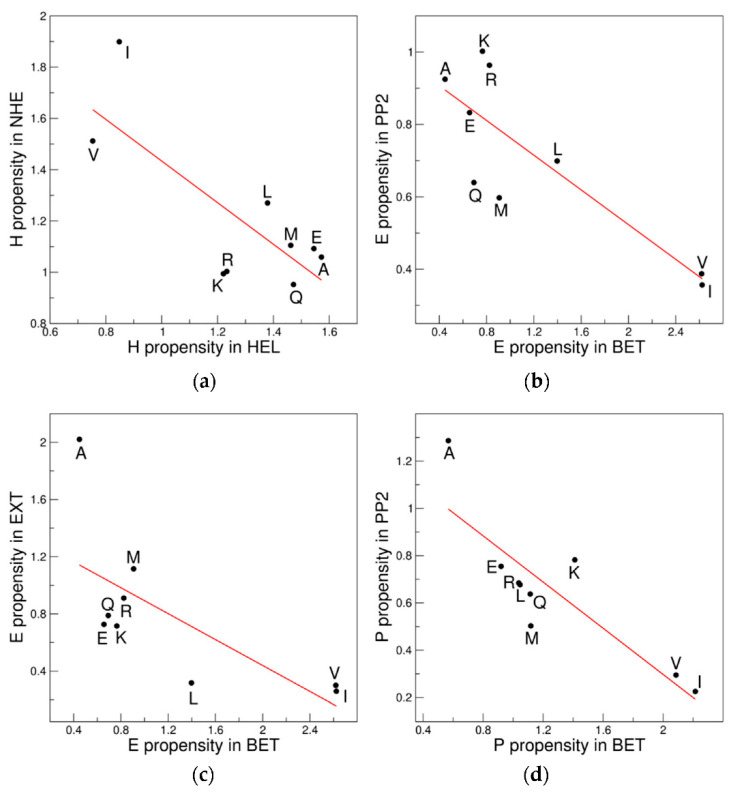
Significant anticorrelations of the propensity scales for the same secondary structure elements in distinct (φ, ψ) clusters detected in the 9AA ensemble: (**a**) HEL_H *versus* NHE_H; (**b**) BET_E *versus* PP2_E; (**c**) BET_E *versus* EXT_E; and (**d**) BET_P *versus* PP2_P.

Our novel subdivision of the Ramachandran space in terms of similarity of (φ, ψ) propensity scales has highlighted unexpected heterogeneity in the regions that correspond to the most common structural elements such as helices and sheets. To check the impact on the propensity scales of the (φ, ψ) dihedral angles and of the structural features of each secondary structure, we compared the propensity scales for the same secondary structure element in different (φ, ψ) clusters and, conversely, for different secondary structure elements in the same cluster. As anticipated above, it is worth mentioning that the same secondary structure element could be formed by residues adopting (φ, ψ) angles that fall in distinct clusters. In detail, residues embodied in α-helices may populate both the HEL (201,827 residues) and NHE (55,461 residues) clusters whereas residues belonging to β-sheets adopt the (φ, ψ) angles of three distinct clusters, BET (107,610 residues), PP2 (9011 residues), and EXT (18,514 residues). Although most of the residues forming polypropline II structures are concentrated in the PP2 cluster (6229 residues), some of them can also populate the BET cluster (2905 residues). Surprisingly, significant anticorrelations could be observed when we compared the propensity scales for the same secondary structure element in (φ, ψ) clusters whose propensity scale are anticorrelated: HEL_H *versus* NHE_H (R = −0.78, *p* = 0.01), BET_E *versus* PP2_E (R = −0.84, *p* = 0.0042), BET_E *versus* EXT_E (R = −0.69, *p* = 0.038), and BET_P *versus* PP2_P (R = −0.86, *p* = 0.003) (Figure 8). Similarly, since residues with similar (φ, ψ) values can form different structures we compared the propensity scales for distinct secondary structure elements in the same cluster. In detail, the cluster HEL is populated by residues forming either α-helix (201,827 residues) or 3 (10)-helix (20,378 residues) structures. The regions corresponding to the well-known β-strand and polypropline II structures (BET and PP2) are not strictly confined but tend to overlap. Residues classified as E or P by DSSP can populate, although to different extents, both the BET (107,610 residues in E and 2905 residues in P) and PP2 (9011 residues in E and 6229 residues in P) clusters.

**Figure 9:**
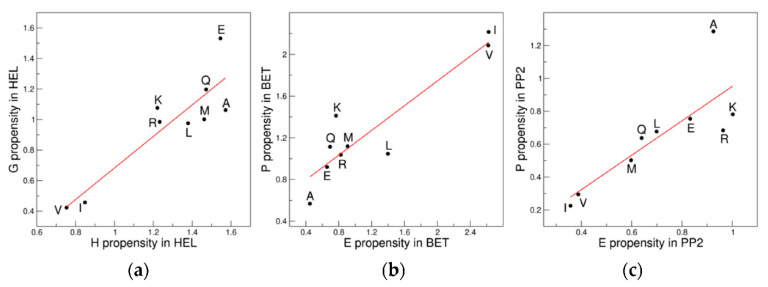
Significant correlations of the propensity scales for different secondary structure elements in the same (φ, ψ) clusters detected in the 9AA ensemble: (**a**) HEL_H *versus* HEL_G; (**b**) BET_E *versus* BET_P; and (**c**) PP2_E *versus* PP2_P.

Comparing the propensity scales for the two helical structures (H and G) in the same cluster (HEL), we observe that the significant ‘H *versus* G’ correlation previously detected (R = 0.87, *p* = 0.0024) for the 9AA ensemble in the Ramachandran plot is confirmed or it is even more significant in this cluster (R = 0.89, *p* = 0.0013) (Figure 9). Surprisingly, the propensity scales for the β-sheet and polypropline II structures (E and P), which, as previously shown, anticorrelate (R = −0.69, *p* = 0.038—9AA ensemble) if the 9AA residues populating the entire Ramachandran space were considered, are instead strongly correlated if calculated in the individual BET (R = 0.92, *p* = 4.4 × 10^−4^) and PP2 (R = 0.81, *p* = 0.0081) clusters (Figure 9).

These data clearly show that the propensity scales for different secondary structure elements calculated in the same (φ, ψ) cluster correlate whereas propensity scales of the same secondary structure element in different clusters may anticorrelate, thereby suggesting that propensity scales are primarily influenced by the (φ, ψ) values and not by other specific features of the generated structural motif. The trends observed in both the helical and extended regions for the 9AA are fully confirmed when the ensemble of the eighteen non-Pro/non-Gly residues is considered (Supplementary Figures S6 and S7).

### 3.7. Local Geometry as a Key Factor in Determining the Conformational Preferences of Amino Acid Residues

**Figure 10:**
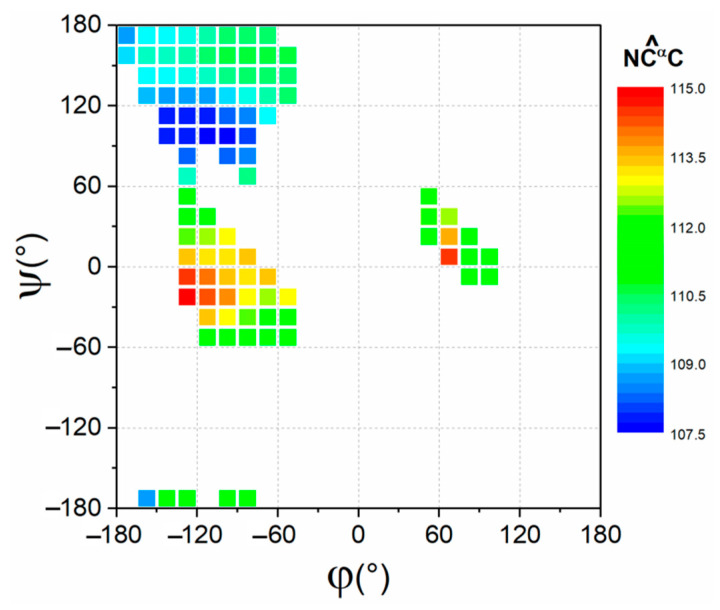
Ramachandran plot highlighting the dependence of the backbone bond angle τ (NC^α^C) on conformation (φ, ψ). The experimental values are calculated in the dataset Data1.6 by averaging the angles of non-Pro/non-Gly residues in the (φ, ψ) boxes. Only boxes containing more than 500 residues were considered.

Among others, our data led to the important observation that residue preferences are strongly driven by the local conformation. Since the dependence of protein backbone geometry (bond angles, dihedral angles and pyramidalization) on local conformation (φ, ψ values) is currently a widely accepted concept in protein structure [36,37,38,39,40,41,42,43,44], we investigated whether (φ, ψ) propensities and local geometry may be related entities. In this framework, the backbone bond angle τ (NC^α^C) represents one of the key points. Thanks to the wide number of high-resolution crystallographic protein structures now available, the relationship between (φ, ψ) torsion angles and τ has been recently extensively studied [36,40,42,44]. Indeed, several statistical and quantum-chemical investigations performed in the last decades have highlighted a combined dependence of this angle on both φ/ψ values [36,40,42,44]. Using a new dataset of well-refined protein structures (Data 1.6, see Methods for further details), we calculated the average value of τ of non-Pro/non-Gly residues in the (φ, ψ) boxes considered in this work (Figure 10). The analysis of the geometry variation, in terms of the backbone bond angle τ, clearly indicates that clusters with rather similar values of the NC^α^C angle present correlated propensity scales, whereas anticorrelations are observed for clusters with very different values of the NC^α^C angle.

**Figure 11:**
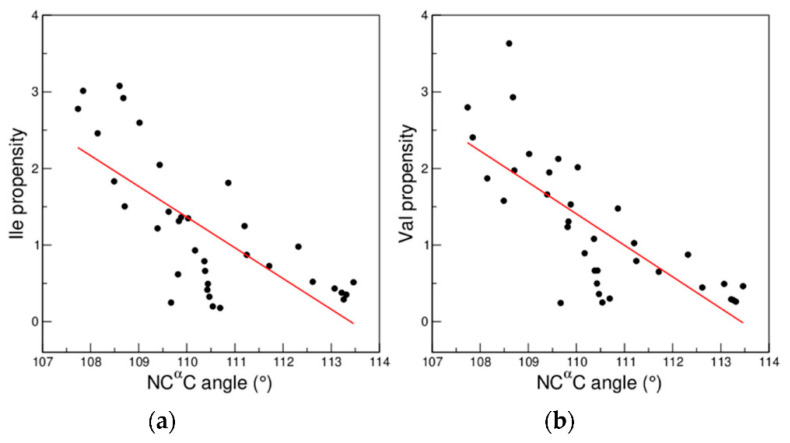
Propensities of (**a**) Ile and (**b**) Val residues as a function of the average value of the backbone bond angle τ (NC^α^C) of the (φ, ψ) boxes. Significant anticorrelations have been detected for both Ile (R = −0.73, *p* < 10^−5^) and Val (R = −0.76, *p* < 10^−5^) residues. Amino acid residue propensities and NC^α^C values were calculated in the dataset Data1.6. Only boxes containing more than 500 residues were considered.

Interestingly, the NC^α^C angles in the BET region adopt significantly lower values than those observed in the nearby regions EXT and PP2 that present propensity scales that are anticorrelated with that of BET. Similarly, the adjacent regions HEL and NHE, which present anticorrelated scales, also display different values of the NC^α^C angle. These qualitative observations were quantitatively assessed by considering the conformational preferences of the branched residues Ile e Val for (φ, ψ) boxes as a function of the average NC^α^C angle of the box as for these residues an increase in NC^α^C is expected to produce an unfavorable local strain [42]. As shown in Figure 11 and Figure S8 we observe a clear decrease in their preference for boxes characterized by larger values of NC^α^C, clearly indicating that the local geometry has an important role in dictating their conformational preferences.

## 4. Conclusions

Since its definition, which dates back to nearly sixty years ago [22], the Ramachandran plot in its many declinations has inspired a remarkable number of insightful studies that have had a tremendous impact on structural biology [23,24,25,26]. Remarkable examples can also be found in the recent literature [45,46,47,48]. We here exploited this tool by initially identifying regions of the plot for which amino acid residues have similar conformational propensities. The comparison of propensity scales computed in these regions clearly indicated that similarities and dissimilarities were not connected to the distance of their location in the Ramachandran plot. Indeed, adjacent regions could display anticorrelated propensity scales. This observation led to the finding that even regions of the Ramachandran plot, such as those corresponding to the α-helix or the β-structure, that are commonly believed to be conformationally uniform may be dissected in regions that are endowed with distinctive conformational propensities. On the other hand, distant (φ, ψ) regions of the Ramachandran plot occasionally exhibit very similar propensities. Our data also indicate that the impact on residue preferences of the local conformation in terms of (φ, ψ) angles is predominant over the local secondary structure. Indeed, propensity scales of residues embodied in different secondary structure elements but adopting the same (φ, ψ) angles are similar. On the other hand, propensity scales based on specific secondary structure elements are different if residues fall in different regions of the Ramachandran space. Although the significance of these findings is very robust for the residues (9AA) that do not form electrostatic, polar or aromatic interactions with their own backbone, similar data emerge from the analysis of the 18 non-Pro/non-Gly residues (18AA). In order to provide an interpretative background to these results, we evaluated the role that the local variability of protein backbone geometry plays in this context. Our analysis indicates that (dis)similarities of propensity scales between different regions of the Ramachandran plot are coupled with (dis)similarities in the local geometry. We here showed that the (φ, ψ) propensities of aliphatic β-branched residues (Val and Ile) clearly anticorrelate with the amplitude of the NC^α^C angle.

Collectively, the present findings provide a solid explanation for the elusive question related to the preference of protein residues for the different conformations of the Ramachandran space. The shape of the side chain of a certain residue, by impacting the local geometry of the backbone may either favor or disfavor the optimal value of the NC^α^C associated with the conformational state. It is important to note that similarities of the propensity scales of the different regions are not dictated by the similarity of the conformation (contiguity in the Ramachandran plot) but rather from the similarity of the NC^α^C angle.

On the basis of the present findings, some literature observations can be easily explained by considering the strain that the side chain imposes on the local backbone geometry. For example, the tendency of Val and Ile to adopt conformations in the polypropline II motif that are distinct from those assumed by other residues [32] is likely related to their attitude to avoid states characterized by large values of the NC^α^C angle. Similarly, the distinctive tendency of Gly residues to adopt conformations with positive values of the φ angle also characterized by ψ values close to zero (Figure 10 of reference [49]) may be attributed to the marginal strain imposed by its side chain on the local conformation, thus endowing this residue with the possibility to adopt states with large values of the NC^α^C angle [49].

These effects here analyzed are clearly evident when residues with aliphatic or long side chains are considered. The presence of functional groups on the residue side chain that can form stabilizing interactions with the backbone atom may interfere with this geometry-based interpretation. Nevertheless, in future studies, using the conceptual framework here developed, the relative impact of these interactions and the local geometry strain can be evaluated to explain the conformational preferences of specific residues for certain regions of the Ramachandran plot.

Finally, being the effects here described independent of the inter-residues interactions and therefore on the (un)folded state of the polypeptide chain they could have a predominant role in affecting the local conformations of intrinsically disordered proteins.

## Figures and Tables

**Table 1:** Pair-wise comparison of the propensity scales for the (φ, ψ) clusters identified in the Ramachandran plot. The correlation coefficients R and the *p*-values are reported on the right and left sides of the diagonal, respectively. The ensemble of nine protein residues (A, V, L, I, M, K, R, Q, E) is considered.

	EXT	PP2	BET	BRI	POS	HEL	NHE
**EXT**		0.90	−0.74	0.45	0.40	0.66	−0.67
**PP2**	0.001		−0.83	0.27	0.40	0.79	−0.73
**BET**	0.022	0.005		−0.59	−0.76	−0.96	0.95
**BRI**	0.22	0.48	0.094		0.72	0.42	−0.70
**POS**	0.28	0.28	0.018	0.029		0.63	−0.82
**HEL**	0.055	0.011	<0.001	0.26	0.069		−0.86
**NHE**	0.048	0.026	<0.001	0.036	0.007	0.003	

**Table 2:** Pair-wise comparison of the propensity scales for the (φ, ψ) clusters identified in the Ramachandran plot. The correlation coefficients R and the *p*-values are reported on the right and left sides of the diagonal, respectively. The ensemble of the eighteen non-Pro/non-Gly residues is considered.

	EXT	PP2	BET	BRI	POS	HEL	NHE
**EXT**		0.64	−0.50	0.38	0.03	0.33	−0.41
**PP2**	0.004		−0.72	−0.08	0.04	0.83	−0.62
**BET**	0.035	<0.001		−0.27	−0.40	−0.83	0.80
**BRI**	0.12	0.75	0.28		0.63	−0.13	−0.37
**POS**	0.91	0.87	0.10	0.005		0.24	−0.67
**HEL**	0.18	<0.001	<0.001	0.61	0.34		−0.76
**NHE**	0.091	0.006	<0.001	0.13	0.002	<0.001	

**Table 3:** Pair-wise comparison of the propensity scales for the (φ, ψ) clusters identified in the Ramachandran plot. The correlation coefficients R and the *p*-values are reported on the right and left sides of the diagonal, respectively. The ensemble of the other nine residues (D, N, C, H, W, T, S, Y, and F) is considered.

	EXT	PP2	BET	BRI	POS	HEL	NHE
**EXT**		0.46	−0.36	−0.18	−0.41	0.13	0.09
**PP2**	0.21		−0.88	−0.52	−0.08	0.88	−0.59
**BET**	0.33	0.002		0.25	−0.25	−0.81	0.64
**BRI**	0.64	0.15	0.52		0.56	−0.51	0.01
**POS**	0.27	0.85	0.51	0.12		0.19	−0.67
**HEL**	0.74	0.002	0.009	0.16	0.63		−0.74
**NHE**	0.82	0.091	0.064	0.99	0.049	0.022	

**Table 4:** Pair-wise comparison of the propensity scales for the secondary structure elements (α-helix (H), 3 (10)-helix (G), β-sheet (E), and polypropline II (P)). The correlation coefficients R and the *p*-values are reported on the right and left side of the diagonal, respectively. The 9AA ensemble is considered.

	E	G	H	P
**E**		−0.96	−0.88	−0.70
**G**	<0.001		0.87	0.63
**H**	0.002	0.002		0.50
**P**	0.038	0.067	0.17	

## Data Availability

Not applicable.

## Supplementary Materials

The following supporting information can be downloaded at: https://www.mdpi.com/article/10.3390/biom12091184/s1, Table S1: PDB codes and some parameters of Data2.2 and Data1.6; Table S2: List of PDB codes of Data2.2 and Data1.6. Table S3: Populations of the (φ, ψ) boxes in Data2.2; Table S4: Populations and propensity scales of (φ, ψ) boxes in Data2.2; Table S5: Average (φ, ψ) values at the seven positions of π-helices; Table S6: Pair-wise comparison of the 18AA propensity scales for the secondary structure elements; Figure S1: Populations of the 576 (φ, ψ) boxes of the Ramachandran plot in Data2.2; Figure S2: Example of calculation of the Chou–Fasman-like propensity; Figure S3: Comparison of the propensity scales of the 9AA; Figure S4: Correlation of the R values; Figure S5: Ramachandran plots; Figure S6: Significant correlations/anticorrelations of the 18AA propensity scales in the helical region; Figure S7: Significant correlations/anticorrelations of the 18AA propensity scales in the extended region; Figure S8: Propensities of Ile and Val residues as a function of NC^α^C.
