# Supplementary material for: Local Backbone Geometry Plays a Critical Role in Determining Conformational Preferences of Amino Acid Residues in Proteins
Source: Biomolecules. 2022 Aug 26;12(9):1184. doi: 10.3390/biom12091184 (PMC9496368; doi:10.3390/biom12091184)
Supplement: Supplementary file 1 [file biomolecules-12-01184-s001.zip › SM/Balasco_SM.pdf]

Supplementary Material

Table S1. PDB codes and some experimental parameters (chain length, resolution, R-factor) of the protein chains of the datasets Data2.2 (a) and Data1.6 (b).

Excel file TableS1.xls

Table S2. List of PDB codes of Data2.2 (a) and Data1.6 (b).

Excel file TableS2.xls

Table S3. Populations of the (φ,ψ) boxes containing at least 1000 residues in the dataset Data2.2. For each box, the number of residues in α-helix (H), 3(10)-helix (G), β-sheet (E), and polyproline II (P)) is also indicated. The total number of residues in Data2.2 is 1,089,468 (265,543 in E; 13,297 in P; 42,006 in G; 376,101 in H).

BOX	tot	E	P	G	H
**1A**	2,218	1,150			
**2A**	6,348	3,550			
**3A**	5,842	3,197			
**4A**	5,010	2,478	1		
**5A**	2,903	921	34		
**6A**	3,019	529	129		
**7A**	6,009	534	376	1	
**8A**	3,939	219	393	3	4
**1B**	2,025	1,179			
**2B**	11,185	7,597			
**3B**	16,369	11,450			
**4B**	16,989	11,318	3		
**5B**	9,086	4,856	129		
**6B**	6,545	2,215	363		1
**7B**	11,205	1,979	1,062	6	1
**8B**	17,984	1,647	2,097	33	9
**9B**	2,421	147	301	12	6
**2C**	6,144	4,216			
**3C**	14,815	11,667			

**4C**	22,848	18,478			
**5C**	17,728	13,685	136		
**6C**	12,066	7,707	401		
**7C**	13,619	5,711	880	5	
**8C**	26,372	5,590	2,359	37	2
**9C**	12,919	1,327	1,077	89	12
**2D**	2,340	1,381			
**3D**	8,739	6,842			
**4D**	24,673	21,355	3		
**5D**	27,966	23,978	127		1
**6D**	19,972	15,234	418	2	2
**7D**	15,980	8,848	691	4	
**8D**	16,345	4,770	1,029	57	1
**9D**	10,044	936	552	150	3
**3E**	2,814	1,736			
**4E**	8,639	6,689			1
**5E**	13,200	10,223	84		
**6E**	12,101	8,147	196		2
**7E**	8,618	4,300	249	2	
**8E**	2,771	751	100	10	1
**3F**	1,195	501			
**4F**	2,602	1,288			
**5F**	3,678	1,920	5		
**6F**	3,744	1,844	11		
**7F**	3,110	1,155	10		2
**4G**	1,858	394		1	
**6G**	1,129	257			
**7G**	2,246	296			1
**4H**	1,726	128		9	
**7H**	2,480	93		2	
**16I**	4,125	333		46	43
**4J**	1,256	42		116	12
**5J**	1,010	33		69	

**16J**	5,725	188		291	29
**17J**	2,851	115		34	16
**4K**	2,204	41		282	68
**5K**	4,524	66		659	37
**6K**	3,355	39		378	6
**16K**	1,220	15		188	1
**17K**	4,884	56		221	13
**18K**	2,234	21		21	1
**4L**	2,311	82		140	303
**5L**	6,632	142		1,251	578
**6L**	12,812	144		2,133	555
**7L**	6,282	62		987	94
**17L**	2,453	15		144	2
**18L**	4,666	45		94	7
**19L**	1,796	16		17	
**4M**	2,047	208		4	562
**5M**	5,003	526		302	1,258
**6M**	10,799	299		1,965	2,079
**7M**	18,634	173		3,446	2,744
**8M**	12,067	77		4,495	1,090
**18M**	2,860	26		31	
**19M**	2,455	21		26	
**4N**	1,196	110			317
**5N**	3,488	552		4	1,069
**6N**	5,704	619		105	1,994
**7N**	13,278	414		868	5,522
**8N**	44,922	208		9,870	19,811
**9N**	9,033	30		3,431	2,204
**5O**	1,696	295			310
**6O**	3,671	732			1,236
**7O**	11,043	732		19	7,171
**8O**	183,473	506		1,692	172,636
**9O**	63,823	113		6,009	51,149

**5P**	1,058	221			80
**6P**	1,785	452			454
**7P**	3,595	388			2,004
**8P**	55,328	212		30	52,919
**9P**	47,610	78		749	45,021
**2X**	1,643	814			
**3X**	1,356	606			
**4X**	1,057	342			
**6X**	1,252	114			
**7X**	1,898	79			

Table S4. Populations (a) and propensity scales (b) of the 95 (φ,ψ) boxes containing at least 1000 residues in the dataset Data2.2.

Excel file TableS4.xls

Table S5. Average (φ,ψ) values at the seven positions of π-helices of the dataset by Karplus PA and colleagues [29].

**Position**	φ (°)	ψ (°)
**PI1**	-64.2	-41.0
**PI2**	-63.3	-47.6
**PI3**	-71.5	-37.9
**PI4**	-91.5	-34.5
**PI5**	-110.5	-52.9
**PI6**	-69.4	-44.9
**PI7**	-64.9	-38.4

Table S6. Pair-wise comparison of the propensity scales for the secondary structure elements (α-helix (H), 3(10)-helix (G), β-sheet (E), and polypropline II (P)).The correlation coefficients R and the *p*-values are reported on the right and left side of the diagonal, respectively. The 18AA ensemble is considered.

	E	G	H	P
**E**		-0.73	-0.27	-0.23
**G**	<0.001		0.12	-0.05
**H**	0.28	0.65		0.59
**P**	0.36	0.83	0.01	

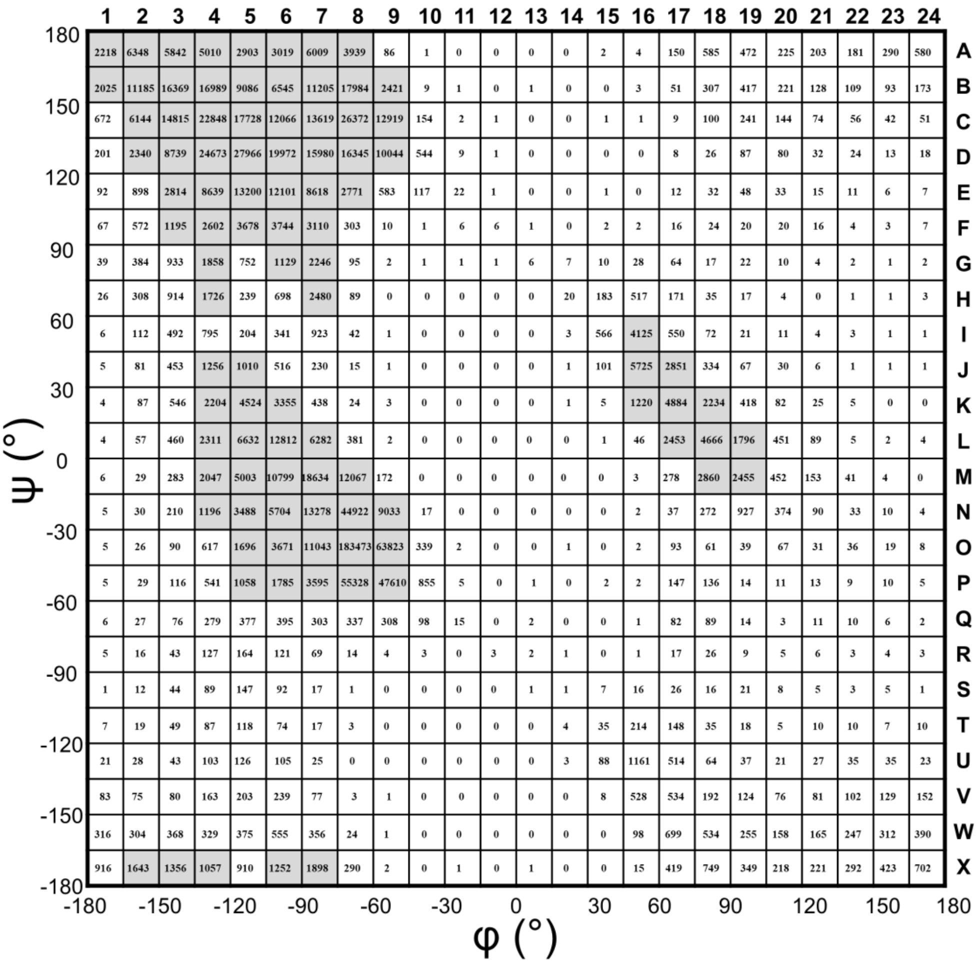

	1	2	3	4	5	6	7	8	9	10	11	12	13	14	15	16	17	18	19	20	21	22	23	24
A	2218	6348	5842	5010	2903	3019	6009	3939	86	1	0	0	0	0	2	4	150	585	472	225	203	181	290	580
B	2025	11185	16369	16989	9086	6545	11205	17984	2421	9	1	0	1	0	0	3	51	307	417	221	128	109	93	173
C	672	6144	14815	22848	17728	12066	13619	26372	12919	154	2	1	0	0	1	1	9	100	241	144	74	56	42	51
D	201	2340	8739	24673	27966	19972	15980	16345	10044	544	9	1	0	0	0	0	8	26	87	80	32	24	13	18
E	92	898	2814	8639	13200	12101	8618	2771	583	117	22	1	0	0	1	0	12	32	48	33	15	11	6	7
F	67	572	1195	2602	3678	3744	3110	303	10	1	6	6	1	0	2	2	16	24	20	20	16	4	3	7
G	39	384	933	1858	752	1129	2246	95	2	1	1	1	6	7	10	28	64	17	22	10	4	2	1	2
H	26	308	914	1726	239	698	2480	89	0	0	0	0	0	20	183	517	171	35	17	4	0	1	1	3
I	6	112	492	795	204	341	923	42	1	0	0	0	0	3	566	4125	550	72	21	11	4	3	1	1
J	5	81	453	1256	1010	516	230	15	1	0	0	0	0	1	101	5725	2851	334	67	30	6	1	1	1
K	4	87	546	2204	4524	3355	438	24	3	0	0	0	0	1	5	1220	4884	2234	418	82	25	5	0	0
L	4	57	460	2311	6632	12812	6282	381	2	0	0	0	0	0	1	46	2453	4666	1796	451	89	5	2	4
M	6	29	283	2047	5003	10799	18634	12067	172	0	0	0	0	0	0	3	278	2860	2455	452	153	41	4	0
N	5	30	210	1196	3488	5704	13278	44922	9033	17	0	0	0	0	0	2	37	272	927	374	90	33	10	4
O	5	26	90	617	1696	3671	11043	183473	63823	339	2	0	0	1	0	2	93	61	39	67	31	36	19	8
P	5	29	116	541	1058	1785	3595	55328	47610	855	5	0	1	0	2	2	147	136	14	11	13	9	10	5
Q	6	27	76	279	377	395	303	337	308	98	15	0	2	0	0	1	82	89	14	3	11	10	6	2
R	5	16	43	127	164	121	69	14	4	3	0	3	2	1	0	1	17	26	9	5	6	3	4	3
S	1	12	44	89	147	92	17	1	0	0	0	0	1	1	7	16	26	16	21	8	5	3	5	1
T	7	19	49	87	118	74	17	3	0	0	0	0	0	4	35	214	148	35	18	5	10	10	7	10
U	21	28	43	103	126	105	25	0	0	0	0	0	0	3	88	1161	514	64	37	21	27	35	35	23
V	83	75	80	163	203	239	77	3	1	0	0	0	0	0	8	528	534	192	124	76	81	102	129	152
W	316	304	368	329	375	555	356	24	1	0	0	0	0	0	0	98	699	534	255	158	165	247	312	390
X	916	1643	1356	1057	910	1252	1898	290	2	0	1	0	1	0	0	15	419	749	349	218	221	292	423	702

Figure S1. Populations (number of amino acid residues) of the 576 (φ,ψ) boxes of the Ramachandran plot in the dataset Data2.2. Boxes containing at least 1000 residues are colored in light grey. Although with a population >1000 residues, the 16U box has not been included in the study being an isolated box in the Ramachandran space.

Residue	box 1A	Data2.2	Propensity
A	203	91,777	1.086
V	1	81,244	0.006
L	27	106,443	0.125
I	2	66,707	0.015
M	21	18,785	0.549
K	43	56,584	0.373
R	77	54,936	0.688
Q	44	40,054	0.540
E	45	66,055	0.335
H	86	25,918	1.630
S	325	63,476	2.515
T	38	61,329	0.304
W	38	17,806	1.048
Y	105	43,721	1.180
N	122	46,277	1.295
D	128	61,317	1.025
C	71	14,364	2.428
F	133	48,764	1.340
G	709	75,312	4.624
P	0	48,599	0.000
TOTAL	2,218	1,089,468	

Example of calculation of the Chou-Fasman-like propensity of the Leucine (L) residue for the (φ,ψ) box 1A, $P_{1A,L}$:

Number of Leucine residues in the box 1A

Number of the 20 aminoacid residues in the box 1A

$$P_{1A,L} = \left(\frac{N_{1A,L}}{N_{Data2.2,L}}\right) / \left(\frac{\sum_{i=1}^{20} N_{1A,i}}{\sum_{i=1}^{20} N_{Data2.2,i}}\right) = \left(\frac{27}{106,443}\right) / \left(\frac{2,218}{1,089,468}\right) = 0.125$$

Number of Leucine residues in the dataset Data2.2

Number of the 20 aminoacid residues in the dataset Data2.2

Figure S2. Example of calculation of the Chou-Fasman-like propensity (see definition (2) in the Methods section) of a certain residue (i.e. Leucine) for a specific box (i.e. box 1A) of the Ramachandran plot.

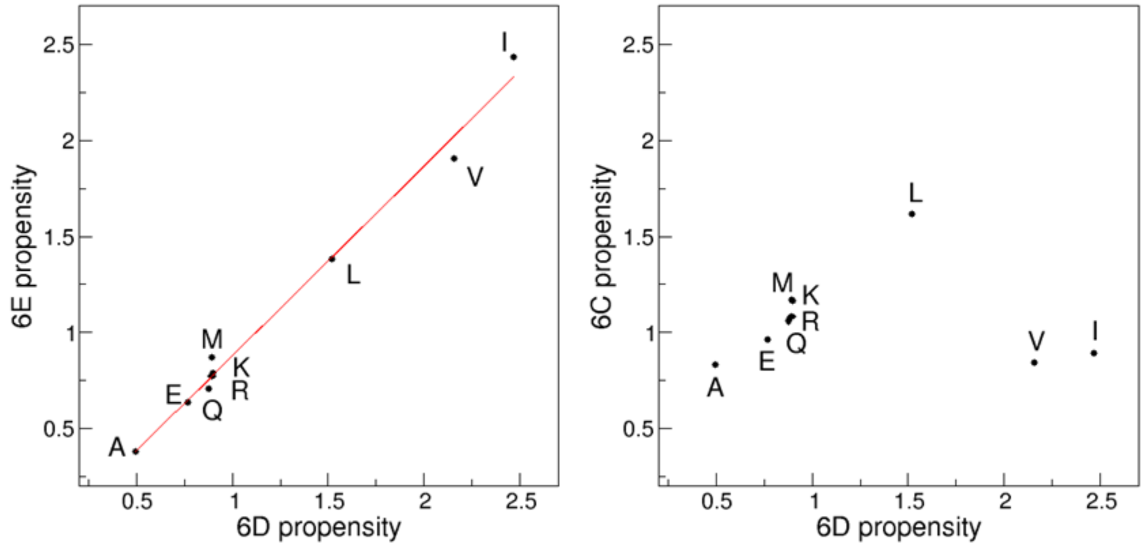

(a) **(b)**

Figure S3. Comparison of the propensity scales of the 9AA for the couples of boxes (a) 6D-6E and (b) 6D-6C. For the 6D-6E pair the linear regression analysis yielded a correlation coefficient R= 0.995 (p-value<10^{-5}).

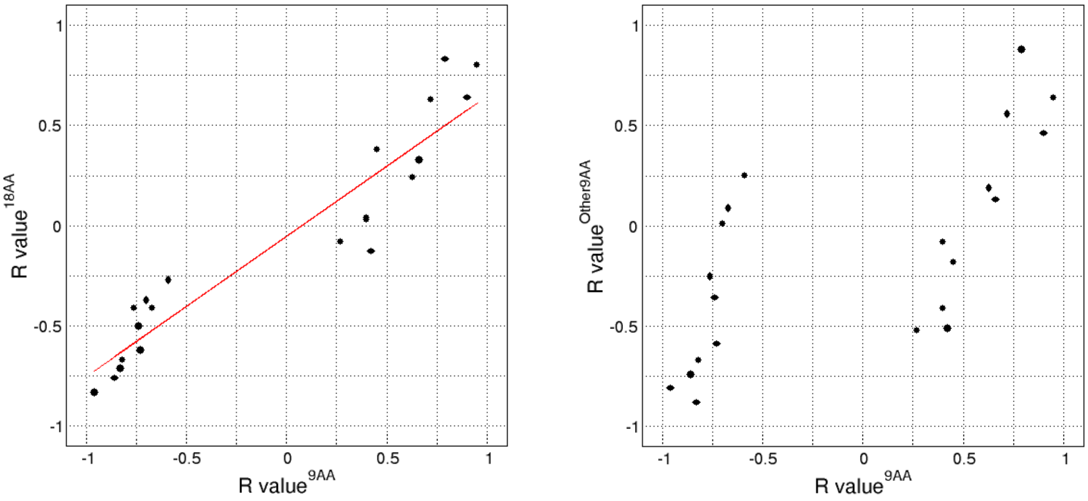

(a) **(b)**

Figure S4. Correlation between the R values obtained for the same pair-wise comparison using (a) the 18AA and 9AA amino acid residue ensembles (R= 0.94, $p<10^{-5}$) and (b) the 9AA and Other9AA ensembles.

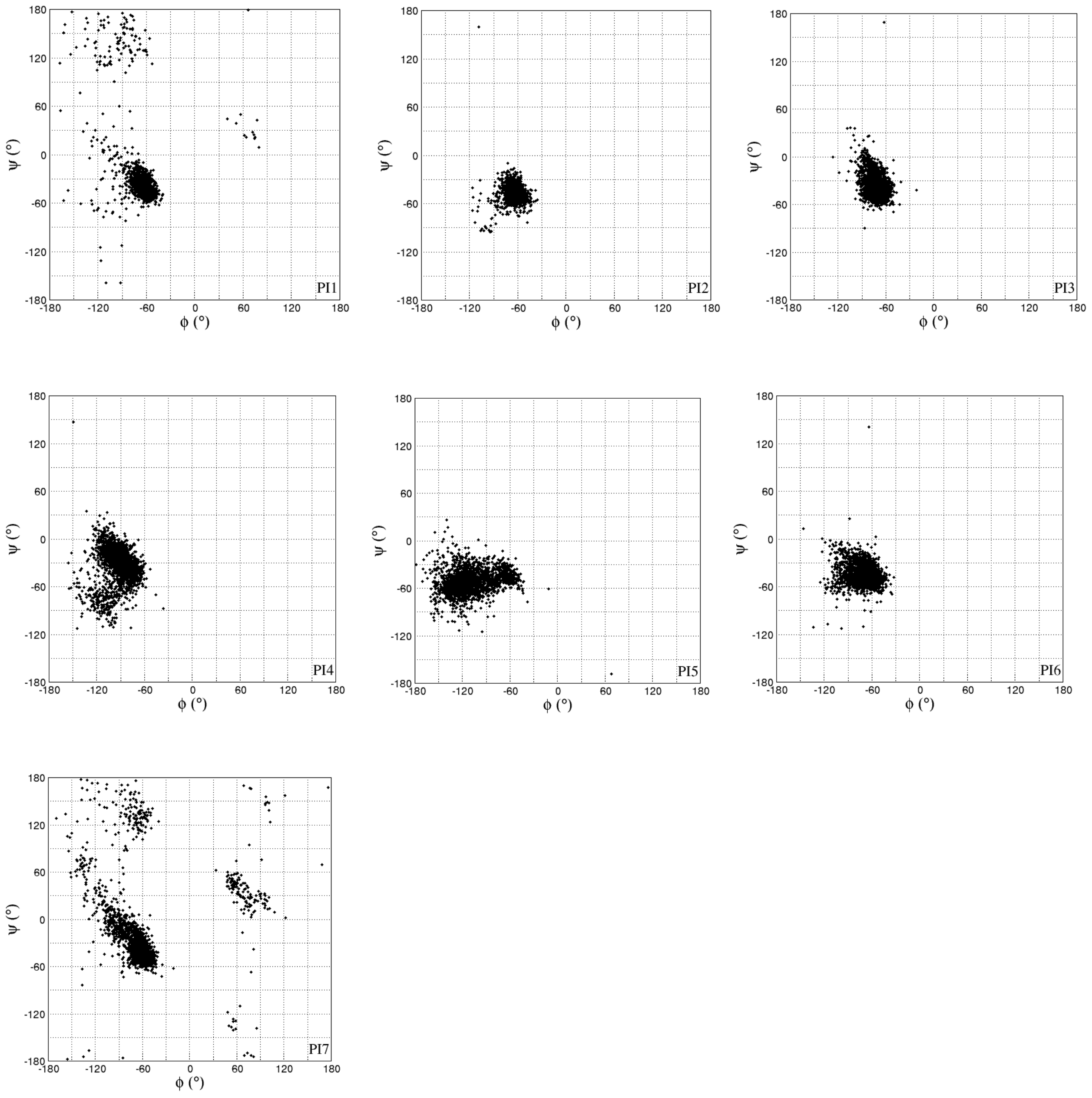

Figure S5. Ramachandran plots showing the conformation adopted by amino acid residues located at the seven positions of π-helices (PI1-PI7).

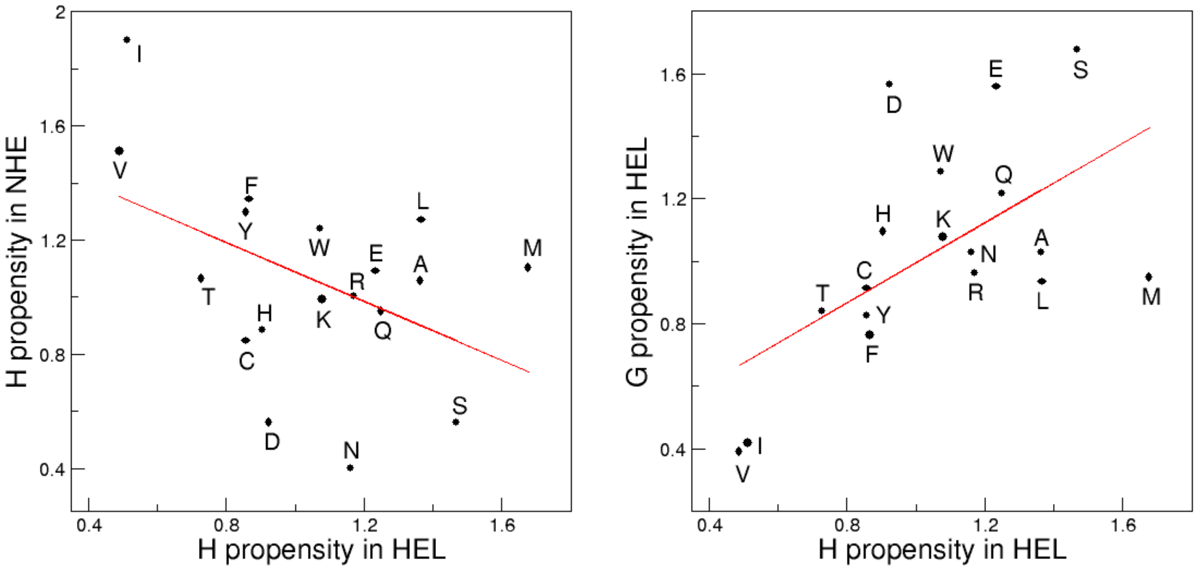

(a) **(b)**

Figure S6. Significant correlations/anticorrelations of the propensity scales detected in the 18AA ensemble: (**a**) HEL_H *versus* NHE_H (R = -0.46, p=0.055), (**b**) HEL_H *versus* HEL_G (R = 0.59, p=0.010).

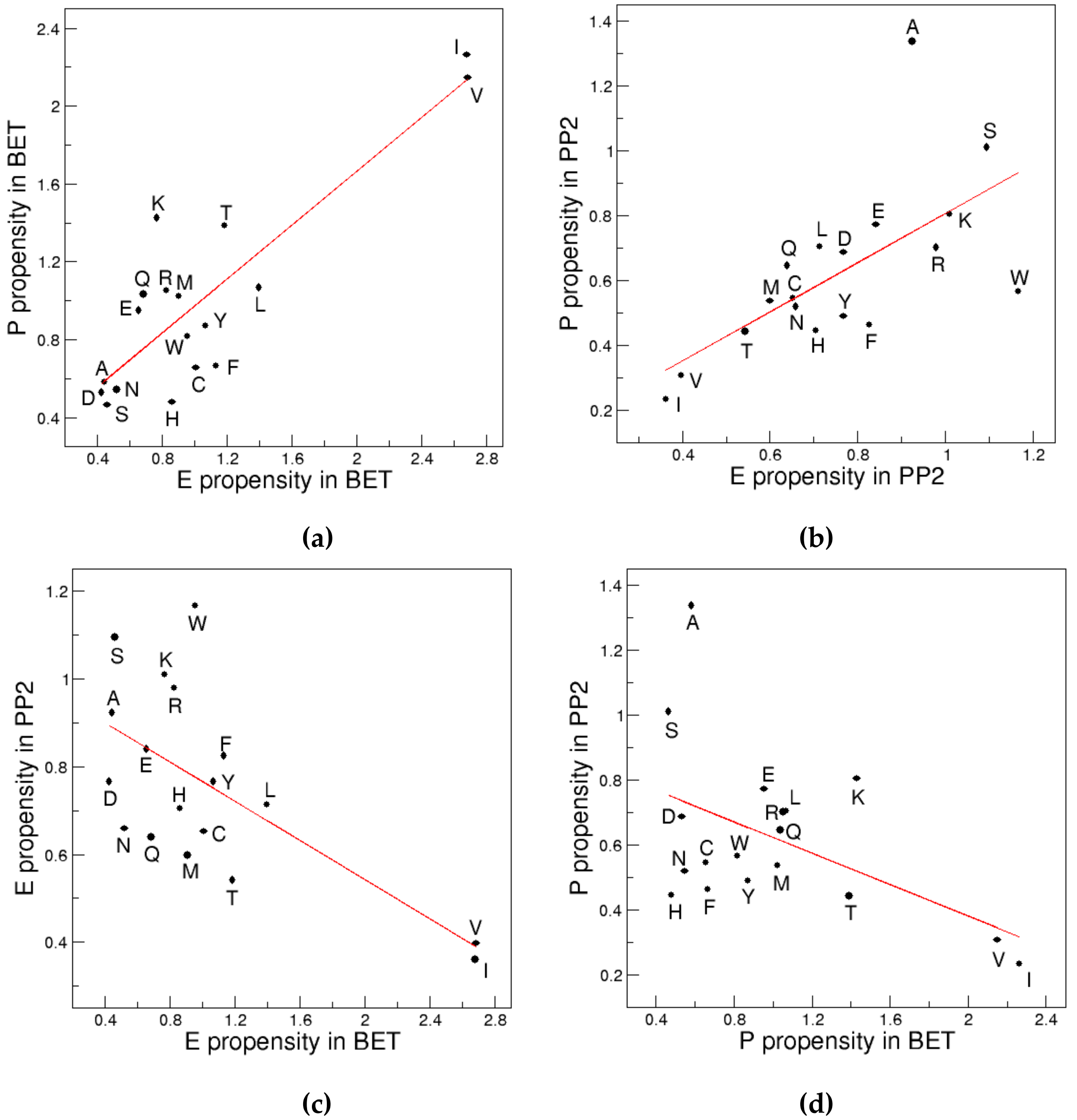

Figure S7. Significant correlations/anticorrelations of the propensity scales detected in the 18AA ensemble: (**a**) BET_E *versus* BET_P (R= 0.87, $p<10^{-5}$), (**b**) PP2_E *versus* PP2_P (R= 0.66, p=0.0029), (**c**) BET_E *versus* PP2_E (R= -0.66, p=0.0029), and (**d**) BET_P *versus* PP2_P (R= -0.49, p=0.039).

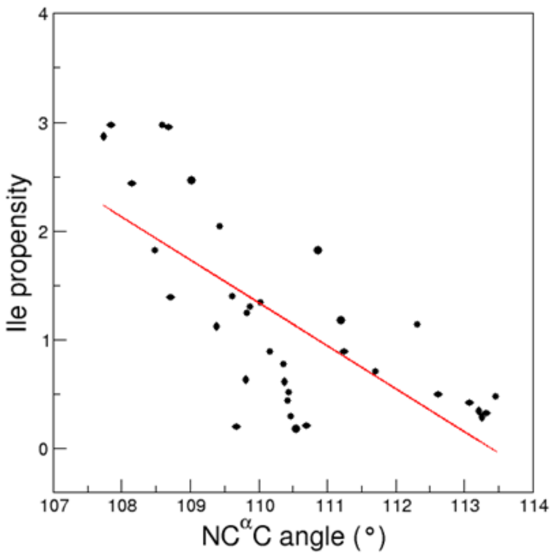

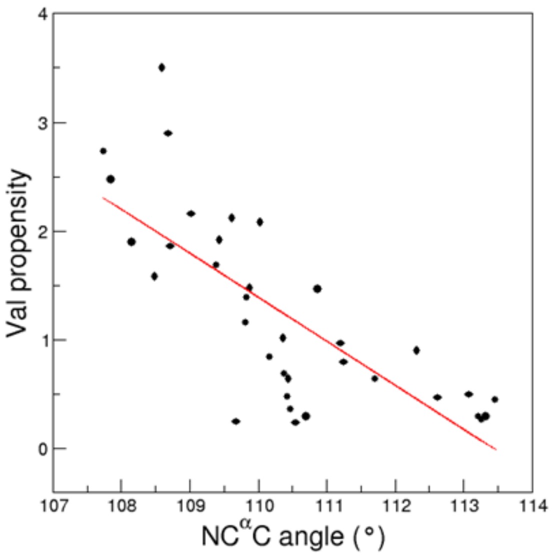

(a) **(b)**

Figure S8. Propensities of (**a**) Ile and (**b**) Val residues as a function of the average value of the backbone bond angle τ (NC$^{\alpha}$C) of the (φ, ψ) boxes. Significant anticorrelations have been detected for both Ile (R= -0.72, p<10^{-5}) and Val (R= -0.76, p<10^{-5}) residues. Amino acid residue propensities and NC$^{\alpha}$C values are calculated in the datasets Data2.2 and Data1.6, respectively.
